# Luminal Flow Amplifies Stent-Based Drug Deposition in Arterial Bifurcations

**DOI:** 10.1371/journal.pone.0008105

**Published:** 2009-12-02

**Authors:** Vijaya B. Kolachalama, Evan G. Levine, Elazer R. Edelman

**Affiliations:** 1 Biomedical Engineering Center, Harvard-MIT Division of Health Sciences and Technology, Massachusetts Institute of Technology, Cambridge, Massachusetts, United States of America; 2 Cardiovascular Division, Brigham and Women's Hospital, Harvard Medical School, Boston, Massachusetts, United States of America; University of Milan-Bicocca, Italy

## Abstract

**Background:**

Treatment of arterial bifurcation lesions using drug-eluting stents (DES) is now common clinical practice and yet the mechanisms governing drug distribution in these complex morphologies are incompletely understood. It is still not evident how to efficiently determine the efficacy of local drug delivery and quantify zones of excessive drug that are harbingers of vascular toxicity and thrombosis, and areas of depletion that are associated with tissue overgrowth and luminal re-narrowing.

**Methods and Results:**

We constructed two-phase computational models of stent-deployed arterial bifurcations simulating blood flow and drug transport to investigate the factors modulating drug distribution when the main-branch (MB) was treated using a DES. Simulations predicted extensive flow-mediated drug delivery in bifurcated vascular beds where the drug distribution patterns are heterogeneous and sensitive to relative stent position and luminal flow. A single DES in the MB coupled with large retrograde luminal flow on the lateral wall of the side-branch (SB) can provide drug deposition on the SB lumen-wall interface, except when the MB stent is downstream of the SB flow divider. In an even more dramatic fashion, the presence of the SB affects drug distribution in the stented MB. Here fluid mechanic effects play an even greater role than in the SB especially when the DES is across and downstream to the flow divider and in a manner dependent upon the Reynolds number.

**Conclusions:**

The flow effects on drug deposition and subsequent uptake from endovascular DES are amplified in bifurcation lesions. When only one branch is stented, a complex interplay occurs – drug deposition in the stented MB is altered by the flow divider imposed by the SB and in the SB by the presence of a DES in the MB. The use of DES in arterial bifurcations requires a complex calculus that balances vascular and stent geometry as well as luminal flow.

## Introduction

Drug-eluting stents (DES) are now the primary choice for percutaneous coronary interventions – implanted in millions of patients [1] and costing billions of dollars [2]. As use extends to non-straightforward lesions and complex geometries, questions abound regarding DES longevity [3] and safety [4], [5]. Moreover, while DES efficacy has never been demonstrated to be dose-dependent, toxicity on the other hand significantly increases with drug concentration [6]. Side-effects rise with amount of drug delivered, absorbed, retained and distributed through the vessel wall in a non-uniform fashion; with apprehension recently compounded by several studies which indicated regions of excessive drug to be more thrombogenic [5], [6]. High grade lesions, lesions with significant clot, inflammation or lipid content are at an even greater risk of stent thrombosis. Similarly, lesions in bifurcations [7], long and tortuous segments [8], and specific vascular beds are particularly vulnerable to disease [5]. As DES are approved only for limited use [9], [10], there is now an urgent need to expand this therapy for broader clinical practice. The question then arises as to whether current revascularization strategies for these straightforward cases using DES can be applied to treat complex atherosclerotic disease as well as inhibit neointimal hyperplastic response and if so, how their effectiveness could be quantified.

Arterial drug distribution patterns become challenging to analyze if the lesion involves more than a vessel such as in the case of bifurcations. Indeed, there is no consensus on best stent placement scenario [11], no understanding as to whether DES will behave in bifurcations as they do in straight segments, and whether drug from a main-branch (MB) stent can be deposited within a side-branch (SB). Bench-top and animal models are at times inadequate as they cannot fully quantify drug pharmacokinetics in complex lesions. In some scenarios, computational modeling offers insight that cannot otherwise be acquired, for example in complex architectures where lesion heterogeneity is compounded by presence of a bifurcation and its associated mechanical environment.

We employed three-dimensional (3D) two-phase steady-state computational models simulating the impact of luminal flow and drug transport through the solid arterial wall after drug delivery from stents deployed in straight or bifurcating arterial vessels. We quantified the spatial heterogeneity in arterial drug deposition for several scenarios of stent placement and luminal flow settings in bifurcated beds. These results provide mechanistic insights as to how drug deposition in a multi-vessel environment is concomitantly modulated by relative stent position, regional blood flow changes due to bifurcation presence, and acute local flow alterations induced by the stent itself.

## Methods

### Geometry modeling and governing equations

**Figure 1:**
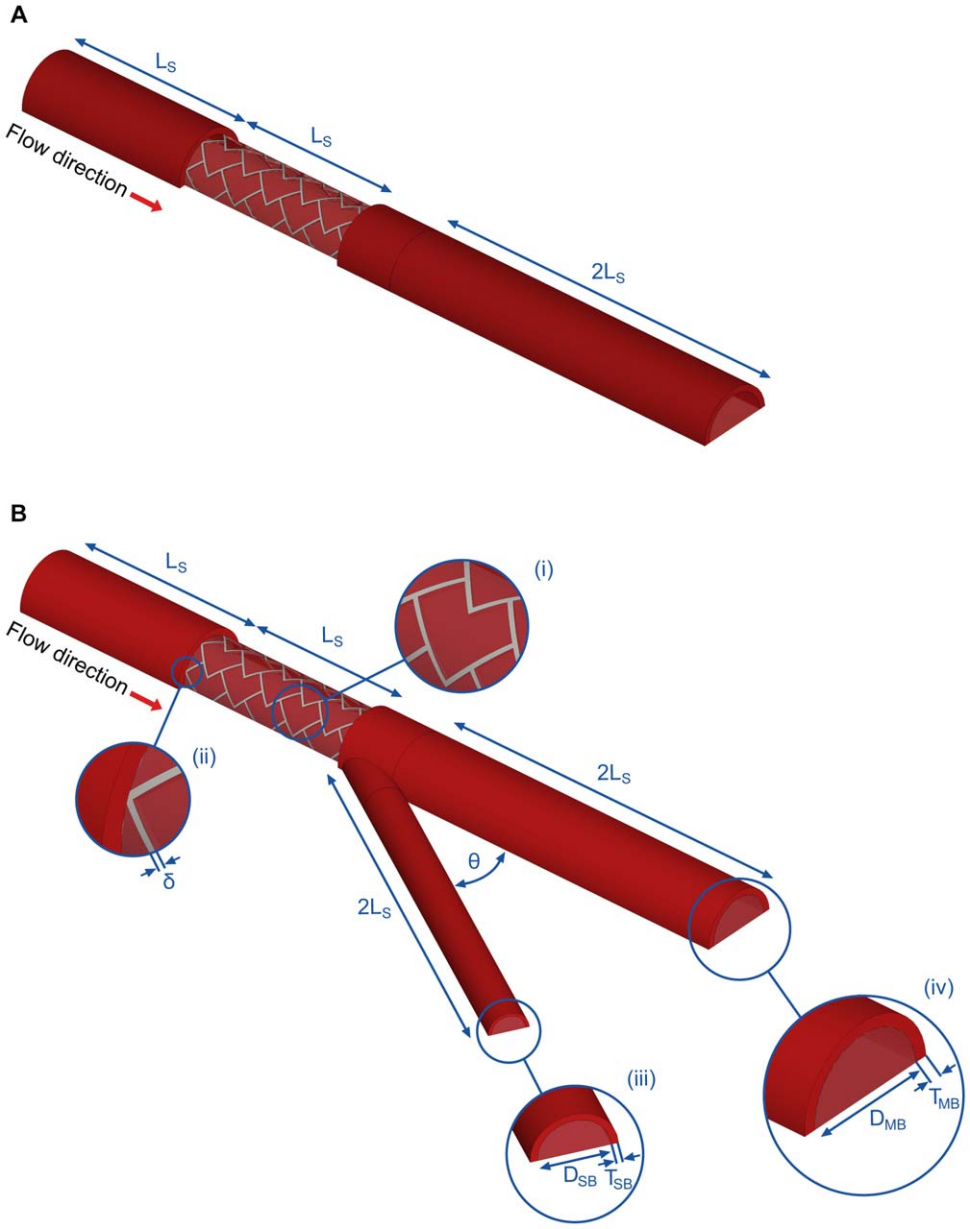
Schematics of the computational models used for the study. A stent of length L_S_ is placed at the upstream section of the arterial vessel in the (A) absence and in the (B) presence of a bifurcation, respectively. Insets in (B) denote delta wing stent design (i), strut thickness (δ) (ii), and the outlets of the side-branch and the main-branch in (iii) and (iv), respectively.

We developed a generalized and automated parametric framework for constructing physiologically realistic three dimensional computational models of single and bifurcated arterial vessels using SolidWorks (Dassault Systèmes) (**Figs. 1A–1B** **, Movie S1**). The geometry generation algorithm allowed for controlled alteration of several parameters including stent location, strut dimensions, stent-cell shape, lumen diameter to arterial tissue thickness ratio, lengths of the arterial branches, extent of stent apposition and the bifurcation angle. For the current study, equal lengths (2L_S_) were assumed for the proximal and distal sections of the MB from the bifurcation. The SB was constructed at an angle of 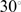. The inlet conditions were based on mean blood flow and diameter measurements obtained from human left anterior descending coronary artery (LAD) [12]. The diameter of the lumen (D_MB_) and thickness (T_MB_) for the MB were defined such that 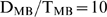 and this ratio was also maintained for the SB.

A DES was placed in different regions of the MB to quantify the effects of stent positioning on arterial drug uptake and one scenario is shown in **Fig. 1B**. A delta wing-shaped cell design [13], [14], [15], [16] belonging to the class of slotted-tube stents was used for all simulations. The length (L_S_) and diameter (D_S_) were fixed at 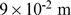 and 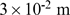, respectively, for the MB stent. All stents were assumed to be perfectly apposed to the lumen of MB and the intrinsic strut shape was modeled as square with length 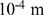. The continuity and momentum equations
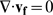
(1)

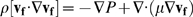
(2)were solved within the arterial lumen, where 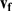,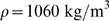, 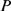 and 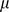 are respectively the velocity, density, pressure and the viscosity of blood. In order to capture boundary layer effects at the lumen-wall (or mural) surface, a Carreau model was employed for all the simulations to account for shear thinning behavior of blood at low shear rates [12], [17].
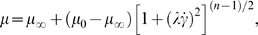
(3)where μ is the effective blood viscosity, 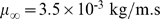 and 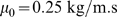 are the blood viscosities at infinite and zero shear rates, respectively, 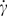 is the shear rate, 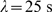 is a time constant, and 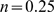 is a power law index.

In the arterial lumen, drug transport followed advection-diffusion process as
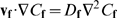
(4)where 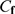 denotes drug concentration within the fluid domain and Paclitaxel was used as a model drug with diffusivity 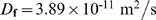 [12], [18]. Similar to the momentum transport in the arterial lumen, the continuity equation was solved within the arterial wall by assuming it as a porous medium.
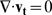
(5)where 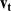 is the interstitial fluid velocity. Further, the momentum equation
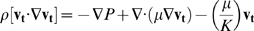
(6)was assumed to follow the Darcy's law where 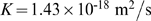 [12], [19] is Darcy's permeability of the arterial wall. An advection-diffusion model was assumed for drug transport within the arterial wall as well.
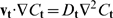
(7)where 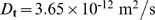 [12], [20] is the model drug diffusivity within the arterial wall and 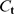 is the concentration of drug in the tissue.

### Boundary conditions

A Poiseuille parabolic velocity profile simulating constant flow condition was imposed at the luminal inlet. At the outlets of the MB and SB, the downstream flow-split was assigned based on the MB and SB outlet areas such that 14% of flow entered the SB. No-slip boundary conditions were imposed on the mural interface and the adluminal surfaces of the stent. Flux continuities of momentum and drug transport were maintained at the mural interface. In the arterial lumen, an open boundary condition for drug concentration was applied at the outlets and symmetry boundary conditions were applied at the flow centerline. An impermeable boundary condition was established at the perivascular aspects of the model vessel. Stent drug release was simulated using a Dirichlet boundary condition of unit concentration.

A finite volume solver (Fluent, ANSYS Inc.) was utilized to perform the coupled flow and drug transport simulations. The semi-implicit method for pressure-linked equations-consistent (SIMPLEC) algorithm was used with second order spatial accuracy. A second order discretization scheme was used to solve the pressure equation and second order upwind schemes were used for the momentum [21] and concentration variables [12]. An under-relaxation factor of 0.8 was used to guarantee smooth convergence of mass transport residuals. All the numerical simulations were performed at a constant Reynolds number 
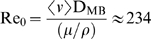
, where the mean velocity was assigned based on the flow and geometry definitions of an average human LAD. Simulations for each case were performed for at least 2500 iterations or until there was a 10^−8^ reduction in the mass transport residual.

### Mesh dependence studies

Mesh dependence studies performed on the non-bifurcating arterial vessel (**Fig. 1A**) at a constant Reynolds number (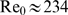) determined the reliability of all subsequent simulations on non-bifurcating vessels. The computational domain composed of tetrahedral control volumes that exploited symmetric vessel characteristics to model half of the arterial vessel. Mesh density was highest in the arterial wall and gradually decreased along the radial direction towards the center line of the arterial lumen. The number of mesh elements on the stent was doubled with successive simulation until convergence of the mass transport residual. Mesh convergence of the constant flow simulations was defined as a less than 2% difference in the volume-weighted average concentration (VWAC) in the entire tissue for two successive mesh refinement iterations. The total cell count in the non-bifurcating vessel after mesh adaption was about 8.9 million. Using the same mesh settings, the fidelity of computer simulations was confirmed for all bifurcated beds by performing mesh dependence studies on a candidate bifurcation geometry (**Fig. 1B**). The resultant cell count for this case was ∼10 million and these settings were used for all the subsequent simulations on bifurcations.

## Results

### Drug distribution in non-bifurcating vessels

**Figure 2:**
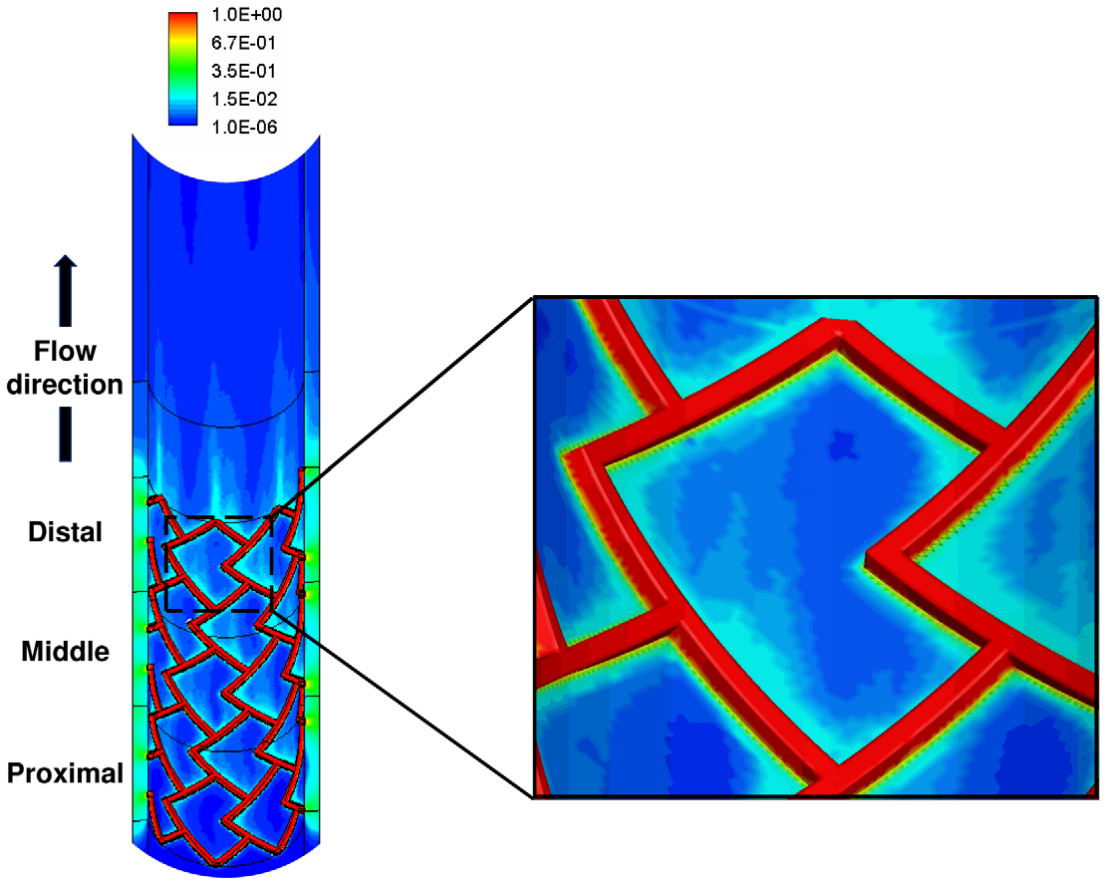
Increased mural drug deposition along the flow direction in a non-bifurcating arterial vessel. Inset shows a high magnification image of drug pattern in the distal stent segment outlined by black dashed line. The entire stent is divided into three equal sections denoted as proximal, middle and distal sections, respectively and the same notation is followed for subsequent analyses.

**Figure 3:**
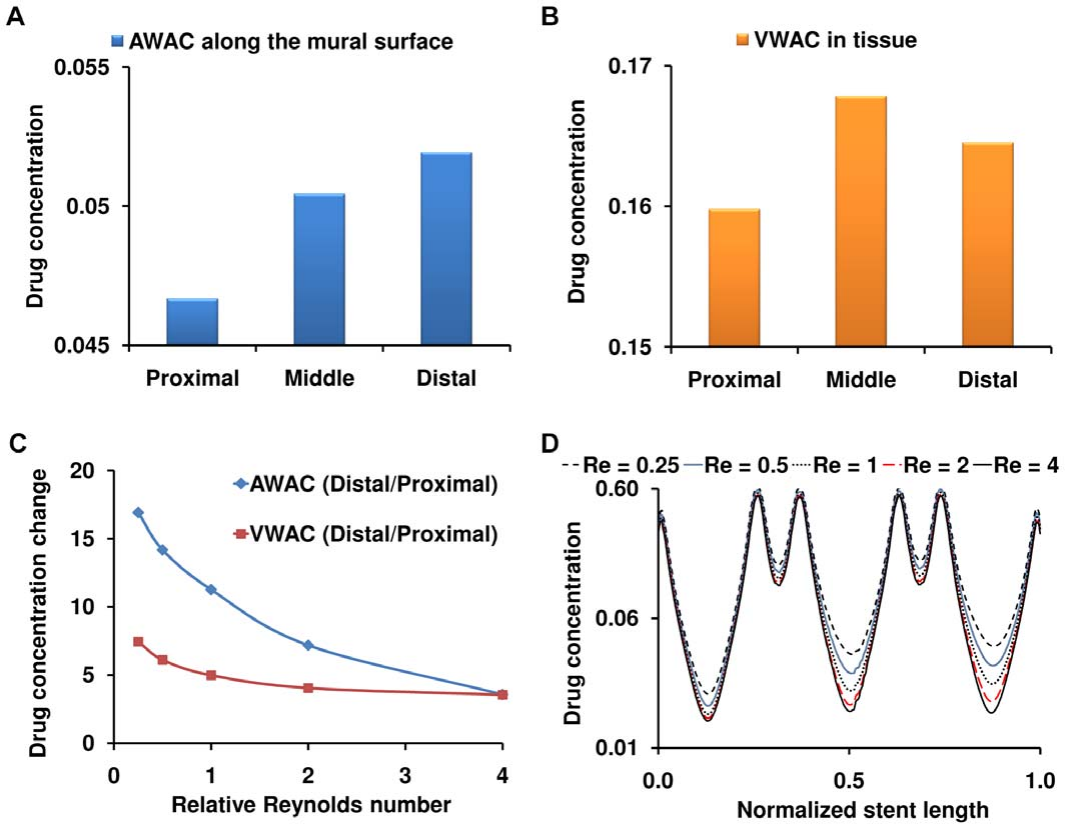
Luminal blood flow decreases spatial heterogeneity in drug deposition. (A) Mural drug deposition denoted as area-weighted average concentration increases along the flow direction. (B) Interestingly, total drug uptake in the arterial wall denoted as volume-weighted average concentration does not vary in the same proportion. (C) Flow-mediated drug deposition quantified as the percentage change between the distal and proximal drug is more evident on the mural surface. (D) Drug concentration at one strut depth below the mural surface beneath the stented region is plotted in log scale indicating that spatial heterogeneity decreases within the inter-strut regions due to increasing flow. All the flow cases in (C) and (D) were normalized using the mean Reynolds number (Re_0_) computed in the Methods section.

Constant flow simulations generate local recirculation zones juxtaposed to the stent which in turn act as secondary sources of drug deposition [12] and induce an asymmetric tissue drug distribution profile in the longitudinal flow direction (**Fig. 2**). Our 3D computational model predicts a far more extensive fluid mechanic effect on drug deposition than previously appreciated in two-dimensional (2D) domains [12], [22], [23], [24]. Within the stented region, drug deposition on the mural interface quantified as the area-weighted average drug concentration (AWAC) in the distal segment of the stent is 12% higher than the proximal segment (**Fig. 3A**). However, total drug uptake in the arterial wall denoted as volume-weighted average concentration (VWAC) is highest in the middle segment of the stent and 5% higher than the proximal stent region (**Fig. 3B**). Taken together, these observations indicate that the flow-mediated effect induced by the presence of the stent in the artery is maximal on the mural surface and increases in the longitudinal flow direction. Further, these results suggest that transmural diffusion-mediated transport sequesters drug from both the proximal and distal portions of the stent into the central segment of the arterial wall beneath the stent. Predicted levels of average drug concentration varied exponentially with linear increments of inlet flow rate but maintained similar relationship between the inter-segment concentration levels within the stented region (**Fig. 3C**).

DES create large drug concentration gradients within the vessel wall [25]. Within each cell, peaks in drug concentration occur precisely beneath the stent and troughs at regions away from the stent surfaces, leading to a spatially heterogeneous drug distribution within the stented region. Interestingly, luminal blood flow decreases the severity of this non-uniformity. Under low flow conditions there is more arterial drug deposition but large concentration gradients prevail along the length of the vessel. However, as flow increases in magnitude, net drug deposition decreases and the concentration gradient along the length of the stent drops (**Fig. 3D**). For the mean flow rate of the LAD denoted as Re_0_, there was about a 50-fold difference between the maximum and minimum drug concentration in the proximal segment of the stent. However, this ratio decreased along the stent length to almost 30-fold in the distal stent segment. These observations imply that not all regions within a stent experience the same drug exposure as convective forces modulate non-uniformity along the stent length. Flow also extends drug distribution to regions beyond the stent deployment site into the downstream segment of the vessel, where the pattern of drug imprint on the mural surface tracks precisely with the proximity of the stent surface (**Fig. 2**).

### Stent position influences drug distribution in bifurcated beds

**Figure 4:**
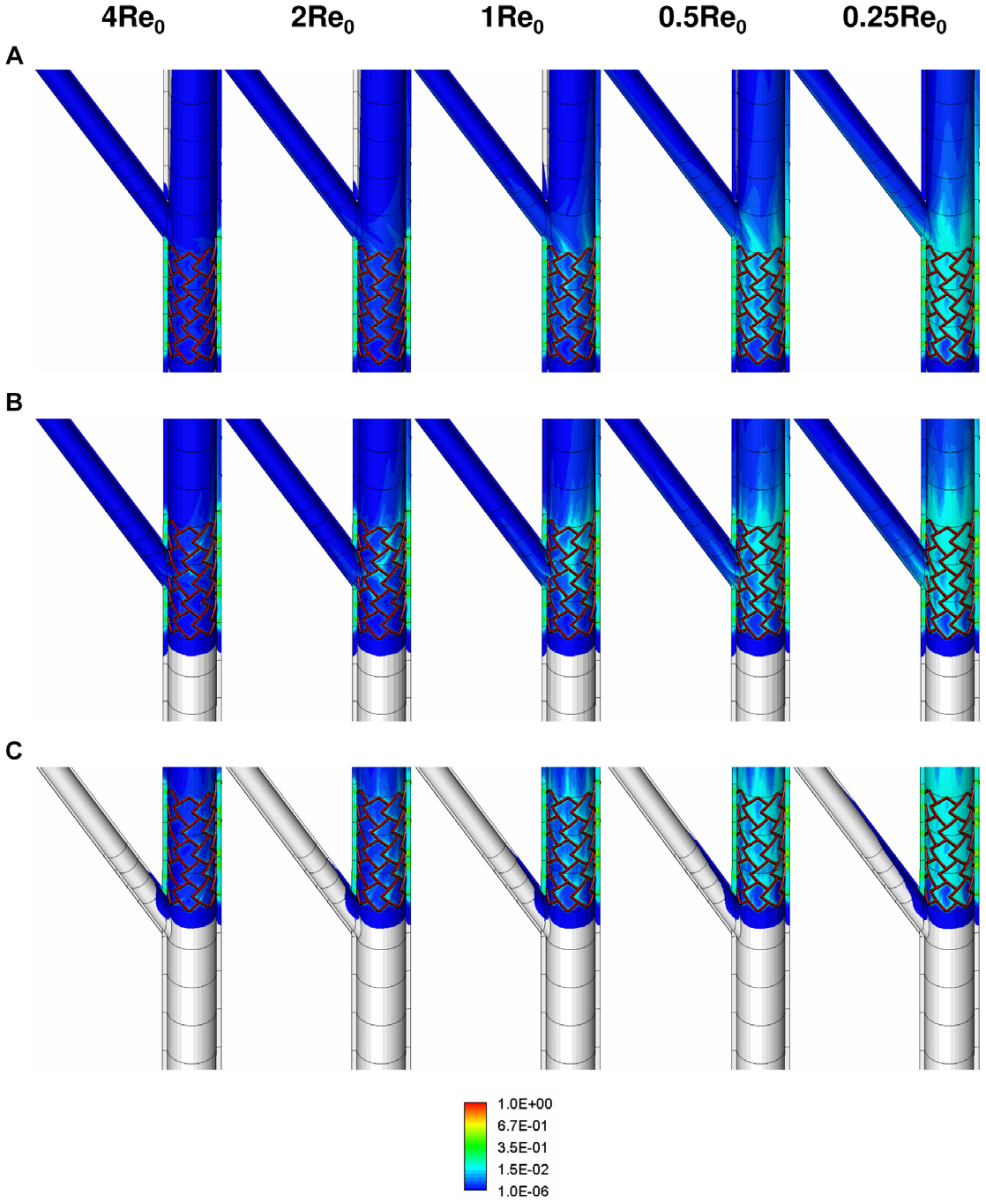
Mural drug deposition is a function of relative stent position with respect to the side-branch and Reynolds number in arterial bifurcations. Snapshots of arterial drug deposition patterns for three different stent placement scenarios (upstream (A), midstream (B) and downstream (C)) and five different flow conditions are shown. All the simulated flow conditions were normalized using the mean Reynolds number (Re_0_) evaluated in the Methods section.

The location of the stent directly modulates the extent to which drug is deposited on the arterial wall as well as spatial gradients that are established in arterial drug distribution. Similar to the non-bifurcating vessel case, peaks in drug deposition occur directly beneath the stent struts regardless of the relative location of the SB with respect to the stent. However, drug distribution and corresponding spatial heterogeneity within inter-strut regions depend on the stent location with respect to the flow divider. In this regard, flow effects imposed by the SB influence drug deposition from a DES in the MB. For example, when a DES is placed within the upstream segment of the bifurcation, blood flow sequesters drug before flow divides at the bifurcation. This solubilized freestream drug acts as the primary source for distal drug deposition. For this case, MB drug distribution within the stented region remains independent of the presence of a bifurcation (Upstream case - **Fig. 4A**). Accordingly, the magnitude and extent of local flow alterations created due to the presence of the stent remain unaltered within the MB stented region and are indistinguishable from the non-bifurcating vessel case. This relationship however changes when the stent is positioned across the bifurcation ostium (Midstream case - **Fig. 4B**) or in the downstream segment of MB beyond the flow divider (Downstream case - **Fig. 4C**). For these cases, the stented portion of the MB overlaps with the boundary layer flow separation and re-attachment region occurring on the lateral wall of the MB across the flow divider. Of note, this flow deranged zone is on a higher length scale than the stent-induced local recirculation zones juxtaposed to each strut and pools drug within the stented region. Nevertheless, it is this combined fluid-mechanic effect that induces circumferential and longitudinal asymmetry in MB drug deposition. At mean Reynolds number Re_0_, the midstream (**Fig. 4B**) and downstream (**Fig. 4C**) cases have about 8% and 15% higher net drug deposition in the MB than the upstream case. Further downstream, MB mural drug deposition becomes more symmetric as flow re-aligns along the longitudinal direction.

The effect of stent positioning on drug deposition seen with mural drug levels extends into the arterial wall and primarily in the zones between the stent struts. Drug deposition beneath stent struts is principally dictated by diffusion and is inviolate with SB-MB stent positioning. There is no predicted difference in drug one strut depth directly below the mural interface but drug in the inter-strut regions varies with respect to the position of the SB relative to the stent. When the SB is upstream of the MB stent there is slower washout and greater amount of drug from the inter-strut regions (**Fig. 4C** **&** **5A**). Mural drug deposition levels remain highest when stents are placed across and within the ostium and downstream segments of MB, respectively (**Figs. 4B, 4C** **&** **5B**).

**Figure 5:**
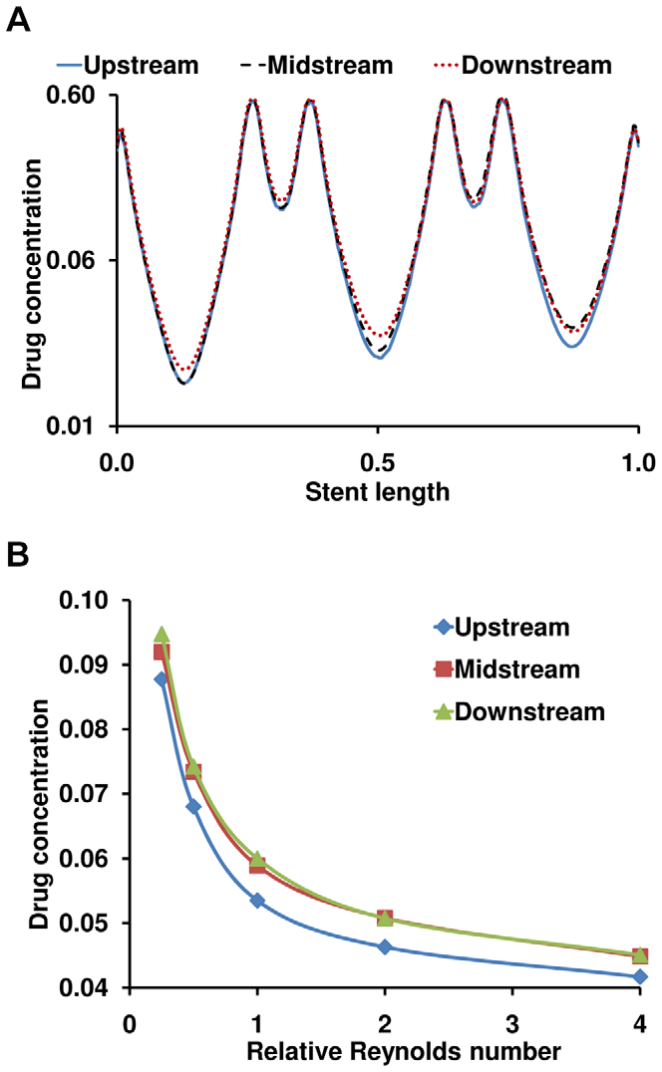
Arterial drug deposition is a function of stent location. (A) Magnitude of drug concentration on the lateral wall of the main-branch within the stented region at one strut depth below the mural surface is shown. All the three cases of stent placement are simulated for the mean Reynolds number (Re_0_) evaluated in the Methods section. Drug concentration is plotted in log scale and the abscissa is normalized with respect to stent length. (B) Changes in drug concentration within the stented region of the main-branch are shown as a function of stent placement and Reynolds number. The abscissa is scaled with respect to the mean Reynolds number (Re_0_) evaluated in the Methods section.

**Figure 6:**
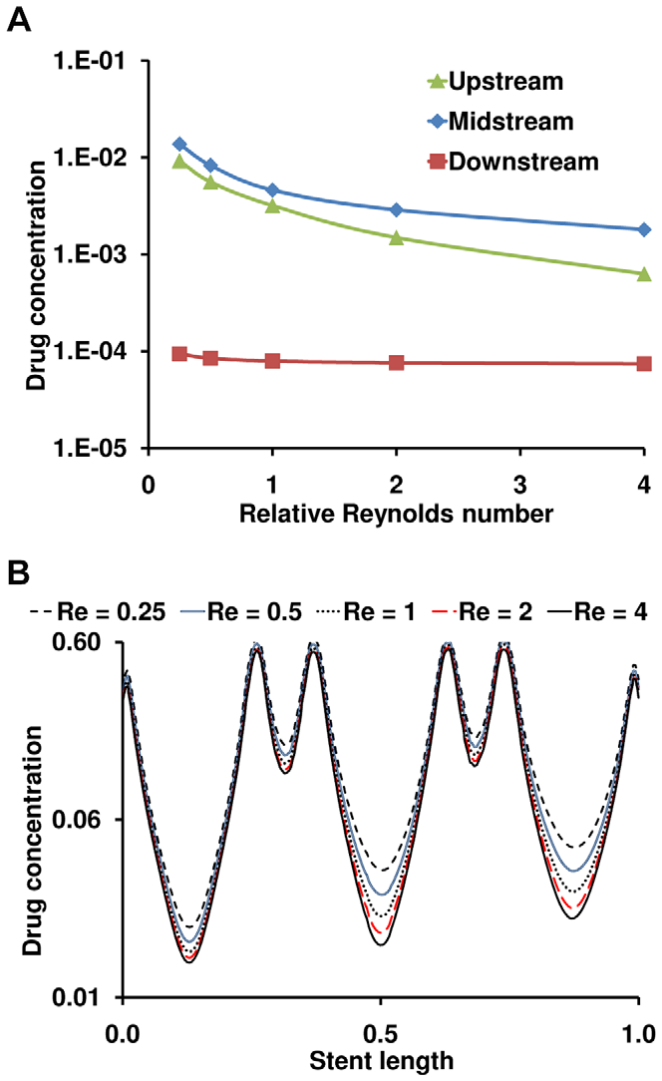
Arterial drug deposition is mediated by flow in bifurcated beds. (A) Drug deposition decreases with increase in Reynolds number regardless of stent placement. Changes in drug concentration within the side-branch are shown as a function of stent placement and Reynolds number. (B) Flow affects inter-strut drug deposition in bifurcated beds. Drug concentration peaks occurring beneath the stent struts are almost unperturbed by flow but the inter-strut regions of the stent are more affected due to alterations in Reynolds number. Note that the stent was placed across bifurcation ostium within the main-branch as shown in Figure 4B. In both plots, drug concentration is plotted in log scale and the abscissa is normalized with respect to stent length and the mean Reynolds number (Re_0_) evaluated in the Methods section, respectively.

Stent positioning also modulates SB drug distribution patterns. Blood flow through all bifurcation geometries creates boundary layer separation and re-attachment on the lateral walls of the SB that pools drug and induces drug deposition. This effect is maximal when stents are placed closer to the ostium and decreases with the stent proximity to the flow divider (**Fig. 6A**). Stent placement in the MB distal to the flow divider has an almost negligible effect on SB drug distribution. As but one example, for the Re_0_ case there is 40- and 58-fold higher drug deposition within the upstream segment of the SB when a stent is placed either proximal (Upstream case) or across the ostium (Midstream case), respectively, as compared to the case with a stent placed in the distal segment of MB (Downstream case). These proportions gradually decrease with increase in net luminal flow as Reynolds number increases the resistance to flow into the SB.

### Impact of flow on drug distribution in bifurcations

One can now appreciate how blood flow and flow dividers affect arterial drug deposition, and especially on inter-strut drug deposition (**Figs. 4B & 4C**). Drug deposition within the stented region of MB (**Fig. 5B**) and the entire SB (**Fig. 6A**) significantly decreases with flow acceleration regardless of stent placement. Relative positioning still dominates but AWAC within the MB region underwent a maximal 2.2-fold for all the placement cases for a 16-fold increase in arterial flow. Flow effects dominate within the inter-strut regions of the stent as they are exposed to varied convective-mediated transport (**Fig. 6B**). Here the higher scale effects of stent strut-induced flow alterations come into play. The struts themselves protrude into and disrupt the flow field inducing flow separation and re-attachment zones that modulate drug release from the adluminal sides of the stent [12]. This effect amplifies the change in drug delivered to and penetrating into the inter-strut arterial segments, raising drug levels in these regions and reducing the range between local maximum and minimum drug concentrations.

## Discussion

Local endovascular drug delivery was long assumed to be governed by diffusion alone [25], [26]. The impact of flow was thought to be restricted to systemic dilution. 2D computational models suggested a complex interplay between the stent and blood flow [12], [22], [23], [24]. Even though struts are orders of magnitude smaller than the vessel dimensions, they are approximately of the same scale as the flow boundary layer. These stent protrusions can obstruct luminal flow, create local flow disruptions, and induce areas of stasis that prolong arterial wall exposure to stent-eluted drug. We now demonstrate that spatial variation in tissue drug uptake can be significant when appreciated from a 3D perspective. Local flow over anisotropic stent designs is subject to heterogeneous flow forces and disruptions leading to asymmetric drug deposition (**Fig. 2**). When the arterial geometry is itself no longer symmetric, additional flow disruptions arise and further asymmetries are detected. The spatial variations in tissue drug that arise from flow alterations can help one appreciate why it has been so difficult to define the most effective percutaneous intervention and optimal utilization of DES in complex arterial geometries. It might now be possible as well to understand the effects of altered lesion morphology and dimensions that occur on a scale greater than stent struts but lesser than the full dimensions of the artery itself.

Bifurcation lesions are an ideal and practical clinical model of great immediate importance for examining the interplay between flow and stent-based drug delivery. Percutaneous interventions on bifurcated vessels are sub-optimal and there is no clinical consensus to their approach. Animal model systems demonstrate that flow disruptions are re-established in certain regions near the flow divider leading to adverse outcomes such as angiographically detectable restenosis patterns [27], and that these flow effects should persist to an even greater extent if intimal hyperplasia is inhibited by DES [28]. Precisely under these circumstances, the role of fluid mechanics is paramount as multiple zones of the bifurcated regime experience flow stasis and subsequent drug pooling.

Fluid dynamic coupling caused by the stent and MB and/or SB vessel dilation precludes quantitative understandings of arterial drug deposition patterns using animal models, let alone in the clinical domain. A computational schema can rigorously consider all relevant determinants of arterial drug distribution, and systematically delineate the relative effects of each variable. The significance of these quantitative findings is enhanced when one can extend the lessons learned in certain domains where other modes of analyses are limited. Previous studies focusing on simplified computer models of stent-implanted arterial vessels explained that intrinsic stent design and associated freestream luminal flow modulate arterial drug distribution [12], [24]. Indeed, the extension of our model from 2D to 3D demonstrated an even greater local deposition differential than was previously appreciated. This and other 2D studies paved the way for moving from modeling of idealized DES and physical systems to physiologically realistic scenarios which can propel the understanding of DES therapy for complex atherosclerotic disease.

The principal aim of this study was to harness the power of computer modeling and focus particularly on bifurcated regimes. Here the MB patency and associated fluid mechanics were altered under specific DES intervention settings to realize a significant dependence of drug concentration levels within the SB as well as the MB. We demonstrate that coupled hemodynamic alterations due to stenting and inherent presence of a bifurcation always affect arterial drug distribution patterns. Specifically, stent struts create local obstructions to flow and pool drug locally while dilated bifurcation vessels naturally create large retrograde flow regimes that accumulate drug; these phenomena differentially modulate arterial drug distribution patterns. Overall, our physiologically realistic 3D computational model predicts that flow-mediated effects create asymmetry in drug distribution and these impressions are far more extensive in bifurcation regimes and modulated by relative stent position with respect to the arterial flow divider and the mean Reynolds number entering the bifurcated regime (**Figs. 4A–4C**).

### Pre-clinical and clinical applications

Computational models are increasingly used to predict regrowth of treated lesions [29], examine device safety [30] and correlate device efficacy with in vivo response [31]. Further, this quantitative methodology can cost-effectively guide the pre-clinical animal and bench-top testing of cardiovascular devices. Predicted maps of drug distribution patterns can guide post-processing of pre-clinical studies and define regions of interest for detailed histologic examination in tissue samples. The lateral upstream segment of a bifurcation SB and MB wall segment opposite to the flow divider will be of particular interest in understanding flow-mediated drug delivery from stents. Further, computational results can direct clinical trial investigators to examine specific areas of angiograms and particular clinical events at given points in time and in patient subsets at risk. In both the preclinical and clinical domains, in silico modeling can help design trials pre-hoc and examine specific findings post-hoc.

### Regulatory perspectives

In silico modeling has profound and expanding regulatory applications. As clinicians are confronted daily with identifying optimal bifurcation lesion treatment strategies, the US Food and Drug Administration (FDA) is developing cost-efficient ways of determining the effectiveness of single vessel devices or complex bifurcation stents for regulatory approval. This issue of complexity is becoming all the more acute as the “simple” cases are covered by approvals for previously evaluated technologies [9], [10]. Most of the new submissions to FDA involve DES devices that serve non-straightforward lesions or geometries. In these cases, DES development based on traditional preclinical, animal and human testing has not identified devices that demonstrate superiority over non-DES controls. As a result, investments of substantial time and money have not lead to important approvals for DES in complex atherosclerotic disease. Meanwhile, the combinatorial bulk and data mass have overwhelmed clinicians, investigators, and reviewers, and there is no current consensus on the appropriate stenting strategy in these lesions. Computational models may help unify and advance clinical practice, especially as these procedural complexities rise.

### Study limitations and future directions

Computational methodology facilitated mechanistic understandings of arterial drug distribution patterns under different settings of MB treatment. Our model paves the way forward for selective bench-top testing and animal investigation but does not yet include complexities of lesion morphology and the pulsatile nature of blood flow. Nevertheless, our previous studies [12] demonstrated that the effect of transient flow behavior on arterial drug deposition can be reasonably approximated using steady state flow models.

Our generalized computer-assisted design strategy facilitates the creation of geometry models that can incorporate several stent designs. We presented the hemodynamic effects on drug distribution patterns using a simplified uniform-cell stent design, though our methodology is adaptable to several types of stents with variable design features. Variability in arterial drug distribution due to other geometric and morphologic aspects such as bifurcation angle, arterial taper as well as presence of a trifurcation [32] can also be understood using our computational framework. Further, performance of a candidate DES using other commonly used stenting procedures for bifurcation lesions such as culotte and crush techniques can be quantified based on their resulting drug distribution patterns and this paradigm can also be extended to predict the effectiveness of dedicated bifurcation stents [33], [34].

### Conclusions

Computational methods coupling flow and mass transfer facilitate cost-effective mechanistic insights on the phenomena governing stent-based drug delivery for complex vascular lesions. Arterial drug deposition and distribution patterns in a bifurcated regime are fascinatingly dependent on stent location as well as free stream, and local retrograde flow. Careful consideration of these insights in conjunction with relevant experimental investigation may lead to superior design and favorable stenting procedures for bifurcation lesions. Flow perturbations prevail under all possible modes of revascularization in a bifurcated milieu and therefore should be viewed in concert with relative stent position as important parameters for understanding the mechanisms governing stent-based drug delivery.

## Supporting Information

Movie S1 Construction of the three dimensional computer-aided design (CAD) model of a stent-deployed arterial bifurcation.
(13.39 MB MPG)
